# Decision to delivery interval for emergency caesarean section in Eastern Uganda: A cross-sectional study

**DOI:** 10.1371/journal.pone.0291953

**Published:** 2023-09-27

**Authors:** Teddy Apako, Solomon Wani, Faith Oguttu, Brendah Nambozo, Doreck Nahurira, Ritah Nantale, Assen Kamwesigye, Julius Wandabwa, Stephen Obbo, Kenneth Mugabe, David Mukunya, Milton W. Musaba

**Affiliations:** 1 Department of Nursing, Faculty of Health Sciences, Busitema University, Mbale, Uganda; 2 Department of Community and Public Health, Faculty of Health Sciences, Busitema University, Mbale, Uganda; 3 Department of Obstetrics and Gynecology, Faculty of Health Sciences, Busitema University, Mbale, Uganda; 4 Department of Obstetrics and Gynecology, Mbale Regional Referral Hospital, Mbale, Uganda; 5 Mbale Regional Referral Hospital, Mbale, Uganda; 6 Department of Research, Nikao Medical Center, Kampala, Uganda; 7 Busitema University Center for Maternal, Reproductive and Child Health, Mbale, Uganda; MUHAS: Muhimbili University of Health and Allied Sciences, UNITED REPUBLIC OF TANZANIA

## Abstract

**Introduction:**

The decision to delivery interval is a key indicator of the quality of obstetric care. This study assessed the decision to delivery interval for emergency cesarean sections and factors associated with delay.

**Methods:**

We conducted a cross-sectional study between October 2022 and December 2022 in the labor ward at Mbale regional referral hospital. Our primary outcome variable was the decision to delivery interval defined as the time interval in minutes from the decision to perform the emergency caesarean section to delivery of the baby. We used an observer checklist and interviewer administered questionnaire to collect data. Stata version 14.0 (StataCorp; College Station, TX, USA) was used to analyze the data.

**Results:**

We enrolled 352 participants; the mean age was 25.9 years and standard deviation (SD) ±5.9 years. The median (interquartile range) decision to delivery interval was 110 minutes (80 to 145). Only 7/352 (2.0%) participants had a decision to delivery time interval of ≤30 minutes. More than three quarters 281 /352 (79.8%) had a decision to delivery interval of greater than 75 minutes. Emergency cesarean section done by intern doctors compared to specialists [Adjusted Prevalence Ratio (aPR): 1.26; 95% CI: (1.09–1.45)] was associated with a prolonged decision to delivery interval.

**Conclusion:**

The average decision to delivery interval was almost 2 hours. Delays were mostly due to health system challenges. We recommend routine monitoring of decision to delivery interval as an indicator of the quality of obstetric care.

## Introduction

Globally, 810 women die to pregnancy related complications and over 6,700 newborns die every day [1, 2]. Sub-Saharan Africa accounts for 70% of global maternal deaths and 43% of global newborn deaths [1, 3]. In Uganda, maternal mortality ratio is 284 deaths per 100,000 live births [1] while the neonatal mortality rate is 19 deaths per 1,000 live births [4]. This is higher than the Sustainable Development Goal (SDG) target of less than 70 maternal deaths per 100,000 live births and 12 neonatal deaths per 1,000 live births [5]. The reported 24-fold difference in intrapartum-related neonatal mortality between high-income and low-income countries shows that improving the quality of obstetric care can effectively reduce maternal and perinatal mortality [6]. Over 22,000 intrapartum related maternal deaths and 1.9 million perinatal deaths that occur annually could be prevented by improving the quality of obstetric care in LMIC [6].

Decision to delivery interval is a key indicator of quality of obstetric care and affects perinatal outcomes [7, 8]. Decision to delivery interval is the duration from time when the decision to birth a baby by emergency cesarean section to the time the baby is birthed [9, 10]. The world Health Organization (WHO) recommends a decision to delivery interval of 30 to 75 minutes depending on the context [11]. However, data from developing countries shows almost 30% of emergency caesarean sections were performed within 30minutes of the decision to deliver [12].

Mbale regional referral hospital is a high volume facility with 12,000 births per year and of these 35% are by caesarean sections [13]. Out of every 1,000 women who under-go an emergency caesarean section in Mbale regional referral hospital, 102 experience a perinatal death. A possible explanation for the high perinatal deaths in Mbale regional referral hospital and other similar health facilities in low and middle- income countries could be poor obstetric care. One of the recommended indicators of the quality of obstetric care is decision to delivery interval. Unfortunately, decision to delivery interval is not routinely documented in low and middle countries,

Therefore, we aimed to assess the decision to delivery interval for emergency cesarean section as a marker of quality of obstetric care at Mbale regional referral hospital.

## Materials and methods

### Study design

We conducted a cross-sectional study between October 2022 and December 2022.

### Study setting

We conducted this study within the labor ward of Mbale regional referral hospital in Eastern Uganda between October 2022 and December 2022. This hospital serves a population of about four million people from 16 districts and one city. Mbale regional referral hospital is the main referral center for four district hospitals and ten health sub-districts in and around Mount Elgon zone. Annually, 12,000 deliveries are registered at Mbale regional referral hospital and cesarean section rate is 35% [13]. It has a capacity of 470 beds. The Obstetrics and Gynecology department has 3 specialists, 2 medical officers, 21 midwives and 8 intern doctors. The labor suite has 6 functional delivery beds and 1 operating theater table and an average of 6–7 emergency cesarean sections daily.

### Study population

Participants were pregnant women who gave birth by emergency cesarean section at Mbale regional referral hospital between October 2022 and December 2022.

### Inclusion criteria

Women at 37 weeks of gestation and above who had to give birth by emergency cesarean section were included in the study.

### Exclusion criteria

Pregnant women admitted in the labor ward with preterm premature labor and needed to deliver by emergency caesarean section as well as women with mental illness were excluded from the study.

### Operational definitions

#### Decision to delivery interval

Time from decision of the emergency cesarean section to delivery of the fetus [14].

#### No delayed decision to delivery interval

Is a decision to delivery interval of less than or equal to 75 minutes.

#### Prolonged decision to delivery interval

Is decision to delivery interval of more than 75 minutes.

### Routine practices in a Ugandan hospital

A woman with labor-like pains is admitted by a midwife at the labor suit. The midwife triages, examines and monitors the progress of labor. A team of medical doctors and specialists do a ward round every morning to review mothers in labor and assess for need to deliver by caesarean section. The decision to deliver by caesarean section is made by the most senior doctor on the ward round with consultation from an available senior midwife. Notes are made in the patient’s file by the junior doctors attending the ward round. Time and date of decision to deliver by caesarean section is documented. The junior doctor obtains consent from the mother for the operation. The midwife on duty prepares the patient for the caesarean section. Preparation involves blood grouping and cross matching, inserting urinary catheter, administering intravenous fluids, pre-operative antibiotics.

The anesthesia team reviews the woman preoperatively. The woman is wheeled to theater as soon as theater space is available. In the theater, the anesthetist documents the various times on an anesthesia chart. The different times include:—time of administration of the anesthetic agent, time of start of the operation, time of skin incision, time of end of operation. Midwife receiving the baby documents the time of delivery of the baby. After the caesarean section, the junior doctor documents the entire procedure of the emergency cesarean section in the patient’s file as dictated by the main surgeon of the operation.

### Study procedure

A research assistant screened mothers for eligibility at the time when a doctor recommended birth by emergency cesarean section in the labor ward. Mothers who were found eligible were consented and enrolled into the study. The research assistant recorded the time when a doctor recommended birth by emergency cesarean section. The mothers were followed up to theatre and the midwife noted the time when the baby was delivered. The decision to delivery interval was determined by calculating the duration between decision to birth by cesarean to the time the baby was birthed. A questionnaire was used to collect information on sociodemographic, obstetric and system factors that may have affected decision to delivery interval.

### Sample size and sampling procedure

We estimated the sample size of this study using Cochran’s Formula for 95% confidence interval, 5% precision and 35.5% of women who had emergency cesarean section within one hour of decision to delivery in Nsambya Hospital in Uganda [15]. This gave us an estimated sample size of 352 participants. Participants were sampled consecutively until the sample size was reached. This involved identifying women sanctioned for emergency cesarean section at the labor suite unit and following them up to when they are taken to the operating theater and later in the in-patient postnatal unit. We defined an emergency cesarean section as a surgery that is conducted when there is an immediate threat to the life of the mother and fetus [16].

### Main variables

#### Outcome variable

The primary outcome was the decision to delivery time interval defined as the time measured from the decision to do an emergency cesarean section to delivery of the fetus [17].

#### Exposure variables

We collected data on socio-demographic factors such as age, marital status, education level, occupation, place of residence, religion, and presence of relatives to give consent. We also collected data on obstetric factors such as ANC visits, previous caesarean section, gravidity, parity, referral status, weight, height, body mass index, and gestational age. Our health system factors included availability of surgical materials and other logistics, type of anesthesia used, time of operation, human resource shortage, busy operating theater, cadre of surgeon and type of anesthesia.

#### Data collection

We developed a questionnaire which consisted of an observational checklist and structured interview questions. This tool developed was piloted on 35 (10%) women who underwent an emergency cesarean section from Namatala health center IV and revised accordingly in order to meet the study objectives before a final copy was sent for approval from the research ethics committee. The consent form was both in English and the local dialect within the study area. Working together with the principal investigator, two research assistants who work in the labor suite as registered midwives of the hospital were trained on the process of data collection and they collected data during day and night.

#### Data analysis and management

The continuous variables were summarized as means and standard deviations or median and inter-quartile ranges as appropriate, while the categorical variables were presented as frequencies and percentages. Data were analyzed using Stata software version 17.0 (StataCorp; College Station, TX, USA) for analysis.

We conducted bivariable and multivariable analyses using a generalized linear model for the Poisson family with a log link to assess the strength of association using prevalence ratios between selected exposures and delay. Variables included in the multivariable model were based on biological plausibility and literature review.

### Ethics

Ethical approval was obtained from Mbale regional referral research and ethical review committee (REF MRRH-2022-211). Written Informed consent was obtained from each of the participants by the participant signing or using their thumbprint before recruitment into the study.

## Results

### Participant characteristics

We enrolled a total of 352 participants with a mean age of 25.9 years standard deviation (SD) ±5.9 years. More than three quarters of the participants 279/352 (79.3%) were aged 20 to 35 years. Majority 247/352 (70.2%) had carried more than one pregnancy, 299 (84.9%) attended at least 4 antenatal care (ANC) visits. More than a third of the participants 131/352(37.2%) were referred from other facilities. Three quarters 273/352 (77.6%) were faced with challenge of lack of surgical supplies before cesarean section. The details are in Table 1.

**Table 1:** Characteristics of women who had emergency caesarean section at Mbale regional referral hospital.

Variables	No delay (n = 71)	Prolonged DDI (n = 281)	Total (352)
**Maternal Age**			
≤19	6(8.5%)	41(14.6%)	47 (13.4%)
20–35	60(84.5%)	219(77.9%)	279 (79.3%)
>35	5(7%)	21(7.5%)	26 (7.4%)
**Gravidity**			
Primigravida	18(25.4%)	87(31%)	105 (29.8%)
Multigravida	40(56.3%)	151(53.7%)	191 (54.3%)
Grand Multigravida	13(18.3%)	43(15.3%)	56 (15.9%)
**Marital status**			
Married	62(87.3%)	244(86.8%)	306 (86.9%)
Single	9(12.7%)	37(13.2%)	46 (13.1%)
**Education level**			
Primary	51(71.8%)	189(67.3%)	240 (68.2%)
Secondary	11(15.5%)	56(19.9%)	67 (19.0%)
Tertiary	9(12.7%)	36(12.8%)	45 (12.8%)
**Occupation**			
Employed	13(18.3%)	54(19.2%)	67 (19.0%)
Unemployed	58(81.7%)	227(80.8%)	285 (81.0%)
**Residence**			
Rural	50(70.4%)	205(73%)	255 (72.4%)
Urban	21(29.6%)	76(27%)	97 (27.6%)
**ANC Visits**			
<4 visits	15(21.1%)	38(13.5%)	53 (15.1%)
≥4 visits	56(78.9%)	243(86.5%)	299 (84.9%)
**Referral status**			
Referred	25(35.2%)	106(37.7%)	131 (37.2%)
Not referred	46(64.8%)	175(62.3%)	221 (62.8%)
**Previous caesarean section**			
No	56(78.9%)	224(79.7%)	280 (79.5%)
Yes	15(21.1%)	57(20.3%)	72 (20.5%)
**Time of operation**			
Day	37(52.1%)	136(48.4%)	173 (49.1%)
Night	34(47.9%)	145(51.6%)	179 (50.9%)
**Day of operation**			
Week day	58(81.7%)	212(75.4%)	270 (76.7%)
Weekend	13(18.3%)	69(24.6%)	82 (23.3%)
**Lack of surgical supplies**			
No	20(28.2%)	59(21%)	79 (22.4%)
Yes	51(71.8%)	222(79%)	273 (77.6%)
**Delay to consent**			
No	67(94.4%)	269(95.7%)	336 (95.5%)
Yes	4(5.6%)	12(4.3%)	16 (4.5%)
**Delay due to no laboratory results**			
No	27(38%)	104(37%)	131 (37.2%)
Yes	44(62%)	177(63%)	221 (62.8%)
**Blood shortage**			
No	57(80.3%)	263(93.6%)	320 (90.9%)
Yes	14(19.7%)	18(6.4%)	32 (9.1%)
**Next of kin**			
Present	71(100%)	280(99.6%)	351 (99.7%)
Absent	0(0%)	1(0.4%)	1 (0.3%)
**Anaesthesia**			
General	17(23.9%)	25(8.9%)	42 (11.9%)
Spinal	54(76.1%)	256(91.1%)	310 (88.1%)
**Cadre of surgeon**			
Intern	27(38%)	194(69%)	221 (62.8%)
Generalist/Specialist	44(62%)	87(31%)	131 (37.2%)

### Decision to delivery interval

The median (interquartile range) decision to delivery interval was 110 minutes (80–145). Only 7/352 (2.0%) participants had a decision to delivery time interval of 30 minutes or less. More than three quarters 281 /352 (79.8%) had a decision to delivery interval of greater than 75 minutes.

### Decision to delivery interval at different times of the day

**Fig 1:**
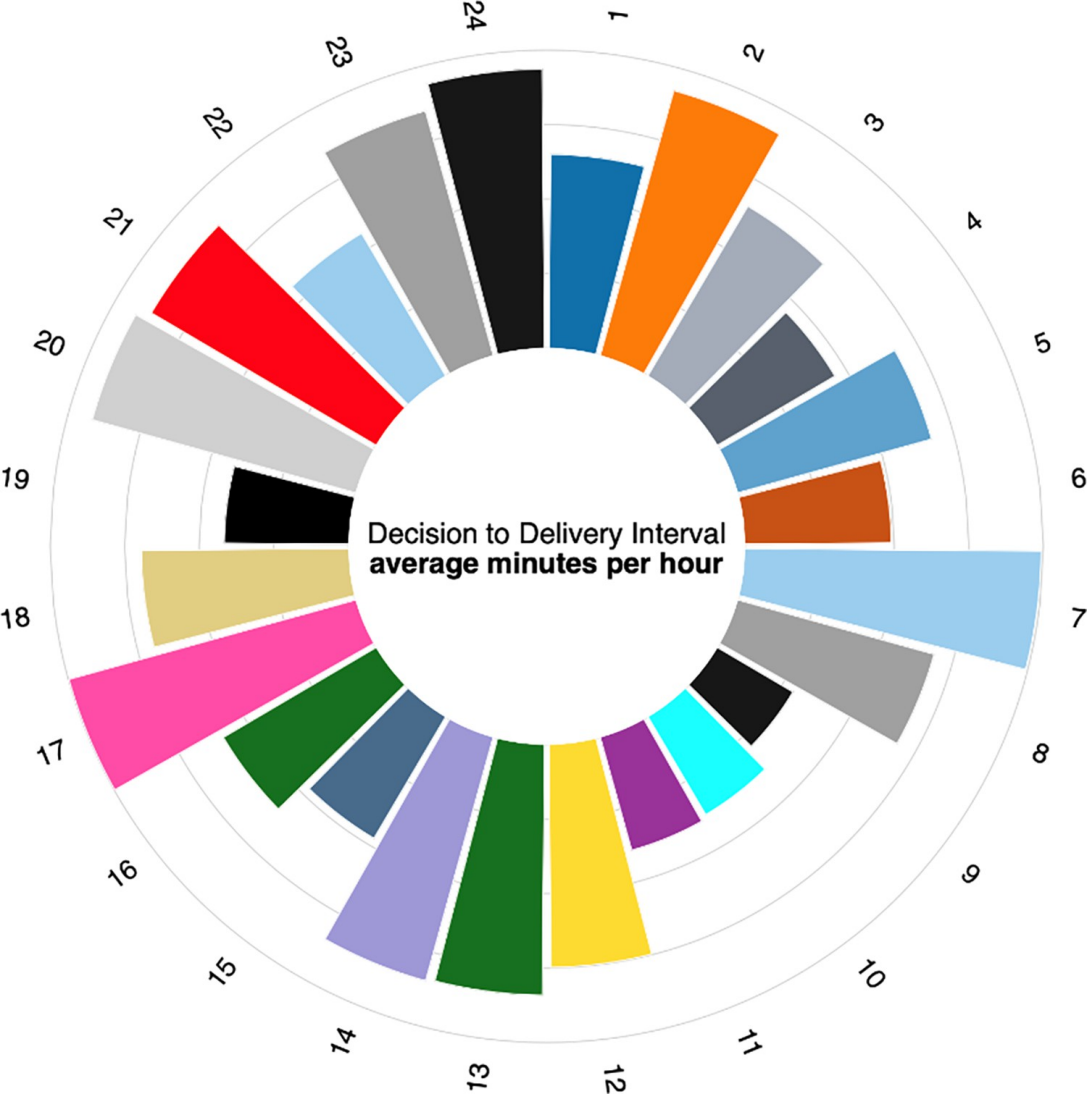
A circular bar plot showing decision to delivery interval at different times of the day.

We also found out that certain hours of the day were associated with a longer duration of decision to delivery time. The day hours (08:00hours to 16:00hours) were associated with less prolonged DDI as compared to the other hours of the day. Particular periods of the day associated with events such as shift change (17:00 hours and between 20:00hrs-21:00 hours), lunch-time (12:00hrs to 14:00hrs) and night sleep (00:00 to 08:00hrs) had longer DDI. This is shown in Fig 1.

#### Indications for emergency caesarean section among women at Mbale regional referral hospital

The commonest indications for emergency caesarean were obstructed labour 163 (46.31%), fetal distress 89 (25.28%), pre-eclampsia 33 (9.38%) and ante partum hemorrhage 26 (7.39%). The details are in Table 2.

**Table 2:** Indications for emergency caesarean section among women at Mbale regional referral hospital.

Indication for emergency caesarean section	No delay	Prolonged DDI	Total
Antepartum hemorrhage	11(15.49)	15(5.34)	26(7.39)
Cord prolapse	1(1.41)	5(1.78)	6(1.7)
Fetal distress	9(12.68)	80(28.47)	89(25.28)
Obstructed labor	29(40.85)	134(47.69)	163(46.31)
Preeclampsia with severe features	9(12.68)	24(8.54)	33(9.38)
Retained second twin	2(2.82)	1(0.36)	3(0.85)
Ruptured uterus	6(8.45)	8(2.85)	14(3.98)
Severe oligohydramnios	4(5.63)	13(4.63)	17(4.83)
Severe polyhydramnios	0(0)	1(0.36)	1(0.28)
Total	71(100)	281(100)	352(100)

### Maternal and perinatal outcomes among women who had emergency caesarean sections at Mbale regional referral hospital

All mothers were alive after the emergency caesarean section. Only 32/352 (9.1%) required blood transfusion. Majority of the babies delivered by emergency caesarean section were alive and well 294/352 (83.52%). Only 20/352 (5.7%) were born dead or were an early neonatal death. The details are in Table 3.

**Table 3:** Maternal and perinatal outcomes among women who had emergency caesarean sections at Mbale regional referral hospital.

Maternal outcomes	No delay	Prolonged DDI	Total
Alive	58(81.69)	262(93.24)	320(90.91)
Alive and got blood transfusion	13(18.31)	19(6.76)	32(9.09)
**Fetal outcomes**			
Alive and in good condition	58(81.69)	236(83.99)	294(83.52)
Alive and transferred to neonatal unit	7(9.86)	31(11.03)	38(10.8)
Dead (early neonatal death)	6(8.45)	14(4.98)	20(5.68)
Total	71(100)	281(100)	352(100)

### Factors associated with prolonged decision to delivery interval

Emergency cesarean section done by intern doctors compared to specialists [Adjusted Prevalence Ratio (aPR): 1.26; 95% CI: (1.09–1.45)] was associated with prolonged decision to delivery interval. The details are in Table 4.

**Table 4:** Factors associated with prolonged decision to delivery interval.

Variable	cPR [95% CI]	P-value	aPR [95% CI]	P-value
**Maternal Age**	** **	** **		
≤19	1		1	
≥20	0.90 [0.80, 1.02]	0.104	0.90 [0.79, 1.01]	0.082
**Maternal Education**	** **	** **		
Primary	1		1	
Secondary/Tertiary	1.04 [0.94, 1.16]	0.447	1.13 [1.00, 1.27]	0.044
**Lack of Surgical Supplies**	** **	** **		
No	1		1	
Yes	1.09 [0.95, 1.25]	0.235	1.11 [0.96, 1.28]	0.149
**Shift**	** **	** **		
Day/Evening	1		1	
Night	1.01 [0.91, 1.12]	0.891	1.01 [0.91, 1.11]	0.875
**Day of Operation**	** **	** **		
Weekday	1		1	
Weekend	1.07 [0.96, 1.20]	0.23	1.07 [0.96, 1.20]	0.217
**Cadre of Surgeon**	** **	** **		
Generalist/Specialist	1		1	
Intern	1.32 [1.16, 1.51]	<0.001	1.26 [1.09, 1.45]	0.001
**Anaesthesia**	** **	** **		
General	1		1	
Spinal	1.39 [1.08, 1.79]	0.012	1.21 [0.92, 1.59]	0.176

## Discussion

We found 80% of emergency cesarean sections having prolonged decision to delivery interval of more than 75 minutes. Hardly any of the emergency cesarean sections were done within the recommended 30 minutes of decision to delivery interval. Less than a quarter had an emergency cesarean section done within the 75 minutes that is deemed acceptable for resource constrained settings. Cadre of the doctor who performed the cesarean section was associated with prolonged decision to delivery interval.

The decision to delivery time in this study was prolonged with a median decision to delivery interval of 110 minutes. This finding of a prolonged decision to delivery interval is not really surprising because similar studies done in Uganda and other Sub-Saharan countries also found the proportion of mothers with a decision to delivery interval of at most 30minutes to be 2% or less i.e. 0.7% in Nsambya, 2% in Mulago, 0.9% in Nigeria 1.7% in Ghana. This may be due to similarities in the health system that is usually characterized by high patient numbers, low staffing and often stock outs of medical supplies needed for emergencies cesarean sections [15, 18–21]. As a referral center with a catchment population of over five million people, the hospital receives a high volume of patients with emergencies from lower health facilities compared to the available health workers and facilities [18, 22]. For instance, the hospital has only one operating table dedicated for emergency obstetric surgeries, which makes it impossible to perform several surgeries at ago. Furthermore, this mismatch between patient volumes and available facilities contributes to the chronic stock out of essential commodities such as drugs and sundries [23]. Lack of surgical supplies further contributes to the preoperative delays even when the theater space is available because patients have to buy them out of pocket.

Decision to delivery interval outside working hours (00:00hours and 08:00hours) was prolonged. This is similar to a study in Germany [24] and can be explained by it being night time when not only the doctor on call is sleeping but also majority of the pharmacies where the surgical logistics are bought are closed. The prolonged decision to delivery interval at 17:00hours and between 20:00hours and 21:00 hours can be attributed to the change in shift whereby there is handover process of the ward that involves various aspects such as report writing, counting of the equipment and patient handover. Furthermore, the prolonged decision to delivery interval at lunch time can be attributed to midwives and doctors spending time having lunch.

Emergency Cesarean sections performed by medical officers and Obstetricians were 26% less likely to have a prolonged decision to delivery interval compared to those by intern doctors. Our results are consistent with findings from India and Ethiopia that reported association between surgery performed by obstetrician and shorter decision to delivery time interval [10, 16]. The possible explanation is medical officers and obstetricians have more clinical experience, quicker decision making unlike the intern doctors undergoing training.

### Strengths and limitations

This is the first study determining decision to delivery time in eastern Uganda in Mbale regional referral hospital.

Our study was limited by the fact that the research assistants and midwives on the ward and in theatre did not have standardized timers or clocks to record the time of decision making and birth of the baby.

We were also unable to accurately measure the theatre waiting time, time taken to give anesthesia and time taken to perform surgery to birth the baby. This was due to shortage of staff and high number of patients attended to by the hospital. Since the study was funded by the principal investigator, only a few research assistants were employed.

## Conclusion

The average decision to delivery interval was almost 2 hours. Delays were mostly due to health system limitations. We recommend routine monitoring of decision to delivery interval as an indicator of the quality of obstetric care.

## Supporting information

S1 Checklist (XLSX)

S1 Dataset (XLS)
